# Facilitators and barriers to accessing hepatitis B care in the postpartum period among foreign-born New Yorkers: a qualitative analysis of case notes

**DOI:** 10.1186/s12889-023-16971-3

**Published:** 2024-01-08

**Authors:** Liz Y. Tang, Farma Pene, Lina Cherfas, Jessie Schwartz, María C. Baquero

**Affiliations:** 1 New York City Department of Health and Mental Hygiene, 42-09 28th Street, Long Island City, NY 11101 USA; 2 A Good Question, 42-09 28th Street, Long Island City, NY 11101 USA

**Keywords:** Perinatal hepatitis B, Telephone patient navigation, Low-cost care to underserved population, Health engagement to immigrant, Health education and awareness, Health services accessibility, Case management, Cultural competency, Medical home, Health department in United States

## Abstract

**Background:**

Approximately 241,000 people are living with hepatitis B in New York City. Among those living with hepatitis B, pregnant people are particularly at risk for elevated viral load due to changes in immune response and require prompt linkage to health care. The New York City Department of Health and Mental Hygiene’s Viral Hepatitis Program implemented a telephone-based patient navigation intervention for people living with hepatitis B in the postpartum period to connect them with hepatitis B care.

**Methods:**

During the intervention, patient navigators called participants to inquire about their past experience with receiving care, available supports, and barriers to care, and worked with them to develop a plan with participants for linkage to hepatitis B care. The information collected during initial assessments and follow-up interactions were recorded as case notes. In this qualitative study, researchers conducted a thematic analysis of 102 sets of case notes to examine facilitators and barriers to accessing hepatitis B care among the intervention participants, all of whom were foreign-born and interested in receiving hepatitis B patient navigation services.

**Results:**

The qualitative analysis illustrated the various ways in which patient navigators supported access to hepatitis B care. Findings suggest that receiving care through a preferred provider was a central factor in accessing care, even in the presence of significant barriers such as loss of health insurance and lack of childcare during appointments. Expectations among family members about hepatitis B screening, vaccination and routine clinical follow up were also identified as a facilitator that contributed to participants’ own care.

**Conclusions:**

This study suggests that while there are numerous barriers at the personal and systemic levels, this patient navigation intervention along with the identified facilitators supported people in accessing hepatitis B care. Other patient navigation initiatives can incorporate the lessons from this analysis to support people in connecting to a preferred provider.

**Supplementary Information:**

The online version contains supplementary material available at 10.1186/s12889-023-16971-3.

## Background

Approximately 241,000 people are living with hepatitis B in New York City (NYC) and many lack access to health care, including the 54% estimated to be undiagnosed [1]. Up to 40% of adults living with chronic hepatitis B infection may die prematurely from liver disease without medical care; however, the progression of hepatitis B infection can be prevented by regular health monitoring and antiviral therapy [2]. Among those living with hepatitis B, pregnant people are particularly at risk for elevated hepatitis B viral load and in need of linkage to care [3–5]. People with chronic hepatitis B infection who are pregnant may be immunocompromised due to immune response changes inherent to pregnancy, and therefore are more likely to have a high viral load. In addition, the increase in liver inflammation after delivery may be influenced by the reactivation of the immune system [5]. This can cause perinatal transmission of hepatitis B and, over time, liver inflammation, which can lead to liver cirrhosis [3, 5]. For these individuals, community-based efforts to educate, screen, vaccinate, and link people to care are vital.

The majority of people living with hepatitis B in NYC are people born outside of the United States (U.S.) who have immigrated from countries where hepatitis B is endemic [6]. People born outside of the U.S. with hepatitis B face specific barriers to accessing all types of health care, such as challenges in securing effective language interpretation services, lack of knowledge of how to navigate services, lack of health insurance, and low cultural responsiveness on the part of the health care system [7–11]. In this context, language and cultural affinity may be important facilitators for linkage to hepatitis B care [9–15]. Culturally tailored messaging may also facilitate linkage to care for foreign-born communities by reducing stigma for individuals and providing hepatitis B education to communities experiencing greater disease prevalence [10, 11, 16, 17].

Among pregnant people with chronic hepatitis B infection identified through NYC surveillance data, 94.5% were born outside of the U.S [1]. With the purpose of improving the health of underserved populations who do not have access to other forms of health insurance, New York State (NYS) offers pregnant people temporary insurance through the NYS Medicaid Prenatal Care Coverage Program, regardless of their immigration status [18]. At the time of this study, this temporary coverage expired two months after delivery, leaving many postpartum immigrant parents without insurance, in addition to the other barriers they face to accessing health care. Without coverage, they may not receive regular monitoring of hepatitis B viral load and liver inflammation, which is vital during pregnancy and after delivery. This places them at elevated risk of rapid disease progression, especially in the postpartum period, and puts their infants at risk for perinatal transmission of hepatitis B [3–5].

In efforts to prevent perinatal transmission of hepatitis B, the NYC Department of Health and Mental Hygiene’s (DOHMH) Perinatal Hepatitis B Prevention (PHBP) Program identifies pregnant people living with hepatitis B through laboratory reporting and conducts interviews during pregnancy and postpartum to support complete infant immunization. To facilitate access to hepatitis B care in the postpartum period, NYC DOHMH’s Viral Hepatitis Program (VHP) developed a telephone-based patient navigation program that reaches out to people identified by the PHBP Program shortly after childbirth to connect them with hepatitis B care and reduce barriers to care. The conceptual framework for this telephone-based patient navigation program is Anderson’s health behavior model as adapted by Yang and Hwang [19, 20]. Their model, which explains health service utilization among foreign-born populations in the U.S., takes into account contextual and structural factors such as government policies and healthcare system access, as well as community- and individual-level factors such as health beliefs, financial resources, and specific health needs. The relationships between these factors in the model informed the design of the telephone-based patient navigation program, which was developed to address the barriers of accessing hepatitis B care and increase follow-up hepatitis B care in the postpartum period.

An evaluation of the telephone-based patient navigation program found that during the course of the study period (July 1, 2016–March 31, 2019), participants were 1.66 times more likely to see a hepatitis B care provider within six months of childbirth than those who did not receive the intervention [20]. While three-quarters of all participants who received the telephone-based patient navigation intervention were successfully linked to care [20], the earlier study did not examine the underlying facilitators and barriers to care that exist for this population, which are important to improve health outcomes and guide future program development. Past qualitative research on HIV testing and linkage to care in immigrant communities has identified perceived barriers such as stigma [21–23], immigration concerns [24], and challenges to accessing health insurance coverage [22, 24]. Some of these are echoed in qualitative findings on potential barriers to hepatitis B screening and care [25–27]. Elucidating these facilitators and barriers is critical to ensure timely delivery of care that can prevent the progression of disease. There has been no research conducted on the perceived facilitators and barriers of hepatitis B care specifically among people with hepatitis B born outside of the U.S. who recently gave birth and are living in NYC. To better understand these factors, we explored qualitative data collected from people with hepatitis B contacted by the NYC DOHMH’s telephone-based patient navigation program in the postpartum period. We sought to identify facilitator and barrier factors at multiple-levels, consistent with Yang and Hwang’s health behavior model [19].

## Methods

The VHP recruited two full-time patient navigators and developed program materials starting in 2016. Two part-time student interns enrolled in a Master of Public Health program and were hired by the VHP also supported the intervention as patient navigators. Patient navigators received training and reviewed program materials on motivational interviewing, patient navigation, and hepatitis B health education before implementing the postpartum telephone-based patient navigation intervention. The PHBP Program referred people living with hepatitis B in the immediate postpartum period who accepted patient navigation services using the NYC DOHMH electronic surveillance system. The program notified those referred to expect a patient navigator’s call and confirmed that the referred individuals had access to a personal phone that belonged to them or a phone they shared with their family. Patient navigators were able to obtain alternative or new phone numbers of these individuals in the NYC DOHMH electronic surveillance system or from their family members, when a phone number was changed. Between February 2017 and March 2019, patient navigators contacted referred individuals to conduct an assessment and connect them to hepatitis B care. The authors used the COnsolidated criteria for REporting Qualitative research (COREQ) checklist to report on the research methods and findings [28].

### Participant enrollment

As part of the study protocol, the patient navigator introduced themselves and their role with the NYC DOHMH at the beginning of the outreach call. They used a DOHMH Institutional Review Board (IRB)-approved standardized telephone consent form for participant enrollment, which included the purpose, description, and expectations of the telephone-based patient navigation program and research study. The patient navigator read the telephone consent form out loud, asked the individual if they had any questions about the intervention and if they were interested in participating in the study. If the individual consented to the intervention and study, the patient navigator recorded the patient navigator’s name, study identification number, participant’s NYC DOHMH electronic surveillance system identification number, and enrollment date on the telephone consent form. The consent procedure was performed in the primary languages of participants. Interpretation services were used when participants spoke a language other than the languages spoken by the patient navigators. Study identification numbers were generated using the patient navigator’s initials and a three-digit enrollment order number. All consent forms were stored in binders and kept in the VHP’s locked, secure filing cabinets.

### Data collection and case notes documentation

Patient navigators used a semi-structured interview form (Appendix A) for initial assessments and follow-up telephone interactions with participants. Initial assessments inquired into participants’ past experiences with receiving care, available supports, and barriers to care, and then developed a plan with participants for linkage to care. During follow-up interactions patient navigators provided hepatitis B and liver health education, aided participants in making medical appointments and securing health insurance, and addressed additional questions or concerns. During the initial telephone assessment, patient navigators established whether participants preferred and consented to communication by text message or email. No personal identifiers or disease-specific information were included in text or email communications. Once a participant attended at least one medical appointment for hepatitis B care, including a hepatitis B blood test, and did not seek further patient navigator support, the participant and the patient navigator discussed terminating the intervention.

The information collected during initial assessments and follow-up interactions constituted the case notes, recorded in English, which were saved in a secure Microsoft Access database. Patient navigators continued to record follow-up interactions as long as participants contacted them for additional supports. The case notes dataset was used in this study to examine facilitators and barriers to accessing hepatitis B care among the postpartum population in NYC. Patient navigators were able to access the hepatitis B test results and demographics of participants using the NYC DOHMH electronic surveillance system.

### Research team

Three authors (L.Y.T., F.P. and J.S.) were employees of the NYC DOHMH’s VHP’s telephone-based patient navigation program and had a primary role in planning, designing, implementing, and evaluating the program. L.Y.T. and F.P. worked as full-time patient navigators for the VHP during the time of the study’s intervention. In addition to English, L.Y.T. is fluent in Chinese (Cantonese, Mandarin) and F.P. is fluent in French and Wolof. Both patient navigators had experience in linkage to medical care prior to the study. J.S. served as a clinical coordinator in the VHP and oversaw the study during the intervention. Additionally, one other NYC DOHMH employee and one external evaluator joined the research team after the intervention. M.C.B. was a doctoral-level epidemiologist in the NYC DOHMH’s Epidemiology Services unit and joined the team to support the qualitative analysis and manuscript development. L.C. who had experience in qualitative research and program evaluation was hired by the NYC DOHMH’s VHP as an external evaluator to support the qualitative analysis. L.Y.T. served as the NYC DOHMH VHP’s telephone-based patient navigation program manager and oversaw this study after the intervention. All authors participated in the evaluation of the program and the development of this manuscript. All researchers in this study are women.

### Data analysis

NYC DOHMH staff obtained approval to collect the data and conduct the subsequent qualitative analysis from the NYC DOHMH IRB. The researcher (J.S.) exported the collected data, including the initial assessment and follow-up case notes, from the Microsoft Access database to an Excel spreadsheet. Two NYC DOHMH staff researchers (L.Y.T. and M.C.B.) reviewed the dataset in the spreadsheet and deidentified the dataset prior to analysis to remove all names, health care provider contact information, insurance information, and any other potentially identifying details. The deidentified dataset was shared with the external evaluator (L.C.) via a secure electronic data transfer portal (BISCOM).

The research team analyzed the data following the general inductive approach to thematic analysis, looking for themes among all the notes and comparatively across categories such as primary language and type of health care facility where participants receive care [29]. First, the research team agreed upon the central research questions. One researcher (L.C.) read through a selection of case notes to identify substantive themes and patient characteristic categories that were relevant to the research questions and developed a draft codebook using Microsoft Word; no a priori codes were used. The draft codebook contained the themes of facilitators and barriers to accessing hepatitis B care and program experiences. The research team approached coding with the goal of identifying facilitators and barriers to care linkage. Facilitators and barriers were categorized to personal and systemic levels. Personal facilitators and barriers involved themes related to finance, family, and other personal situations; for example, “Participant lacks or loses insurance coverage”, “Participant has family support to get care”, and “Participant lacks stable housing.” Systemic facilitators and barriers included themes related to the health care system, such as type of health care facility where participants receive care, “Participant is able to make one’s own appointments” and “Participant experiences poor communication with provider, language barriers.” Codes related to experience with the NYC DOHMH VHP’s telephone-based patient navigation intervention and participants’ questions or concerns covered “Patient navigator made appointment(s) for participant” and “Medication regimen/side effects/treatment/lab work” respectively. The research team reviewed the draft codebook, discussed the interpretation of each theme, and made adjustments according to team members’ reading of the case notes.

Once the codebook was finalized (Appendix B), two researchers (L.Y.T. and L.C.) coded 10 sets of case notes (9.8% of the dataset) using Dedoose qualitative analysis software to determine concurrence. They achieved a high degree of interrater reliability (Cohen’s κ^2^ = 0.82, p < .001; 92.3% interrater agreement) [30]. Subsequently, one researcher (L.C.) coded the remaining 92 sets of case notes using Dedoose qualitative analysis software. Coding involved thoroughly reading the case notes and classifying excerpts based on their content. After coding was completed, the research team examined frequencies of the themes overall and according to categories of participants by language group and by whether they access a primary care provider. An analysis report was developed using Microsoft Word as a precursor to writing this manuscript.

While some case notes offered a great deal of detail, other notes were terser. Therefore, we did not analyze the frequency with which they were mentioned within the case notes and counted each theme only once for each participant. Figures in the results section highlighted the most frequently reported facilitators and barriers, with each theme being counted once per participant regardless of how many times it appeared in the respective set of case notes. Less prominent themes were discussed in the text but not included in the figures. We observed thematic saturation; that is, we arrived at a point in the coding where new ideas were not emerging. We calculated the prevalence of sociodemographic and health care-related characteristics and used chi-square tests or Fisher’s exact tests, as appropriate, to detect significant differences across groups.

### Sample

The telephone-based patient navigation intervention enrolled 433 participants during the study period (July 1, 2016 through March 31, 2019). All participants’ (100%) sex at birth was reported as female; patient navigators did not ask about gender identity. The sampling frame was selected based on the completeness of notes and the researchers’ desire to construct a proportional representation of participants based on cultural and linguistic background that reflected in the full study sample of participants, as follows.

We removed 74 participants from the dataset who did not have follow-up notes, leaving a total of 359 participants (Table 1). We sampled randomly within primary language groups to construct an analytic dataset of 102 (28.4%) participants that included 50% Mandarin and Cantonese speakers of East Asian origin (represented proportionally to the full dataset as 80% Mandarin and 20% Cantonese speakers), 25% French, Wolof, and Yoruba speakers from West Africa, and 25% English speakers (54% of whom were from West Africa and the rest from the Caribbean, Central Africa, Central Asia, Europe, South America, South Asia, and Southeast Asia). None of the participants in the sample were born in the U.S. We reduced the proportion of East Asian participants as compared to the full study sample to include a greater diversity of participants and enable comparison across regions of origin.

**Table 1:** Primary languages of foreign-born participants – New York City, July 1, 2016 – March 31, 2019

Primary language	Number of cases with follow-up notes	Number (%) of participants in analytic sample	Region of origin
Mandarin	185	**41 (40.2%)**	East Asia
English	78	**26 (25.5%)**	Various
French	12	**12 (11.8%)**	West Africa
Wolof	12	**12 (11.8%)**	West Africa
Cantonese	41	**10 (9.8%)**	East Asia
Yoruba	1	**1 (1.0%)**	West Africa
Other languages^a^	30	**0**	Various
**Total**	**359**	**102**	

^a^ The other primary languages spoken by participants in the study were: Arabic, Bengali, Fujianese, Hindi, Russian, Spanish, Taishanese, Tibetan, Urdu, and Uzbek

More than half of the participants (56.9%) in the analytic sample reported that they spoke at least some English. French, Wolof, and Yoruba-speaking participants from West Africa were more likely to speak English than participants from East Asia (p < .001, Fisher’s exact test). Participants in the sample lived in all five boroughs of NYC at the time of the study: 48.0% in Brooklyn, 21.6% in the Bronx, 19.6% in Queens, 8.8% in Manhattan, and 2.0% in Staten Island.

Most participants (70.6%) were insured at the time of initial assessment, followed by 22.5% temporarily insured and 6.9% uninsured. Among the insured, 76.4% had Medicaid and 23.6% had private health insurance. Some participants lost insurance coverage during their time in the intervention. In this study, data of participants’ employment status was not collected and the relationship between insurance coverage and employment was not evaluated. During the initial assessment, 63.7% of participants reported that they had a health care provider for hepatitis B care, 55.9% reported that they had a primary care provider (PCP), and 38.2% reported that the hepatitis B care provider was their PCP. We summarize aforementioned characteristics in Appendix C.

## Results

Participants requested and received varying levels of support from the telephone-based patient navigation intervention. Patient navigators recorded between 1 and 53 encounters with participants, a median of 5 and a mean of 8.7. Fifty-one participants (52.3%) interacted with a patient navigator between two and five times. Participants fell into four groups in terms of their overall experiences in the program. The four groups described as follows were not static or mutually exclusive.

A first group of participants had an established relationship with a health care provider prior to the program, had reliable health insurance, and had been continuously in care for hepatitis B prior to, during, and following their pregnancies. A second group of participants similarly had an established relationship with a health care provider and reliable health insurance but had not accessed hepatitis B care in the postpartum period. Participants in the first and second groups needed minimal intervention on the part of patient navigators. Patient navigators provided health education, reminded the participants to schedule regular follow-up appointments for evaluation, blood tests, and liver ultrasounds, and scheduled appointments for them if requested. Once these participants were on a regular follow-up schedule and stated they had no further questions, their cases were completed.

On the other hand, a third group of participants experienced significant barriers to accessing care: they did not have a health care provider for hepatitis B and/or a PCP, their temporary health insurance was terminated, and they had trouble finding a health care provider, scheduling appointments, and navigating the health care system. In these cases, patient navigators provided more intensive intervention, helping connect participants to low-cost, sliding fee scale services at federally qualified health centers (FQHCs) or public hospitals, facilitating communication between participants and health care providers, making appointments, coaching participants through questions to ask during appointments, and helping participants work through issues such as unstable housing. This usually meant a prolonged period of service, with constant follow-up throughout. Lastly, a fourth group of participants consented to the intervention but later disengaged and could not be reached by patient navigators despite multiple attempts by phone call and text message. Some participants were lost to follow-up after beginning to work through barriers to care with patient navigators, while others were lost to follow-up immediately after the initial assessment.

Some participants moved among the four groups as their circumstances changed during the patient navigation process. For example, some participants who were initially on track to continue in hepatitis B care experienced unexpected setbacks like the loss of health insurance coverage due to their spouse’s change in employment. Nonetheless, most participants (77.5%) were recorded as having attended at least one hepatitis B care appointment during postpartum or had a scheduled future appointment (1.0%).

We identified facilitators and barriers in two primary spheres: the personal and the systemic. The status of family members’ hepatitis care, availability of childcare, competing priorities, perceived importance of hepatitis B care, and participants’ ability to make appointments were considered personal facilitators and barriers. Systemic facilitators and barriers included the availability of preferred health care facilities, care with private providers, establishing a relationship with providers for long-term hepatitis B care, health insurance coverage, and the system of scheduling and reminding appointments at health care facilities. The personal and the systemic facilitators and barriers to hepatitis B care are described in detail below.

### Personal facilitators and barriers to care

One of the personal factors that patient navigators explored with participants was whether their family members were previously screened for hepatitis B, vaccinated for hepatitis B, in care for hepatitis B, or had resolved the hepatitis B infection. Patient navigators assisted in connecting family members in NYC who did not know their hepatitis B status, were susceptible to hepatitis B and unvaccinated, or had been diagnosed with hepatitis B but were out of care to hepatitis B services. For family members living outside of NYC, including those in their countries of origin, patient navigators provided education and encouraged participants to share hepatitis B health promotion messages with their family members and get hepatitis B screening, vaccination, and evaluation. Twenty participants in our analytic dataset identified family members with unknown hepatitis B status or in need of hepatitis B care. Encouragingly, 49 participants reported that their family members had been previously vaccinated, followed up with a health care provider, or had acquired immune control and resolved the hepatitis B infection. We considered this a facilitator to the participants’ own care because it may be an indicator of greater awareness and support of hepatitis B care in the family. Additionally, eight participants referred family members to patient navigators or acquired information on their behalf.

For 27 participants, a notable family-related barrier to care was the unavailability of childcare so that participants could attend appointments. Many participants noted that they did not have family in the US, and their only option for childcare were spouses who were unable to stay home due to work schedules. A few participants were able to bring children to their appointments. Other participants strategized with patient navigators to schedule appointments on days when childcare would be available. Only two participants were unable to attend appointments because of their own work schedules. Three participants mentioned that the lack of stable housing was a barrier to obtaining hepatitis B care.

Fourteen participants did not feel that hepatitis B care was a priority for them or did not think that they needed care. These participants did not get care due to time constraints, had lost their health insurance and did not wish to apply for low-cost or sliding fee scale services, and/or felt healthy and had low viral loads at the time of their most recent tests so did not think regular monitoring with a provider was necessary. In response, patient navigators provided health education and communicated the urgency of regular health care visits, blood tests, and liver ultrasounds to address possible hepatitis B progression and complications in order to encourage participants’ engagement in ongoing hepatitis B care.

### Systemic facilitators and barriers to care

While patient navigators were not able to address all the personal and family-related barriers to care due to participants’ unique personal work and living circumstances, patient navigators worked directly and extensively to address systemic factors. As introduced above, most participants successfully attended at least one postpartum hepatitis B care appointment. We identified three factors that were mentioned as associated with receiving care: having health insurance, having identified a preferred health care facility, and receiving care from a provider in private practice. These factors were interrelated: for example, 16 of the 27 participants who identified a preferred provider received care in a private practice and only 2 of the 27 reported difficulties with scheduling health care appointments.^1^ As expected, participants who were noted as having a PCP, having a hepatitis B provider, or having a PCP that is also their hepatitis B provider were more likely to have successfully received care and were more likely to have stated that they could make appointments on their own, without the patient navigator’s help.

**Fig. 1:**
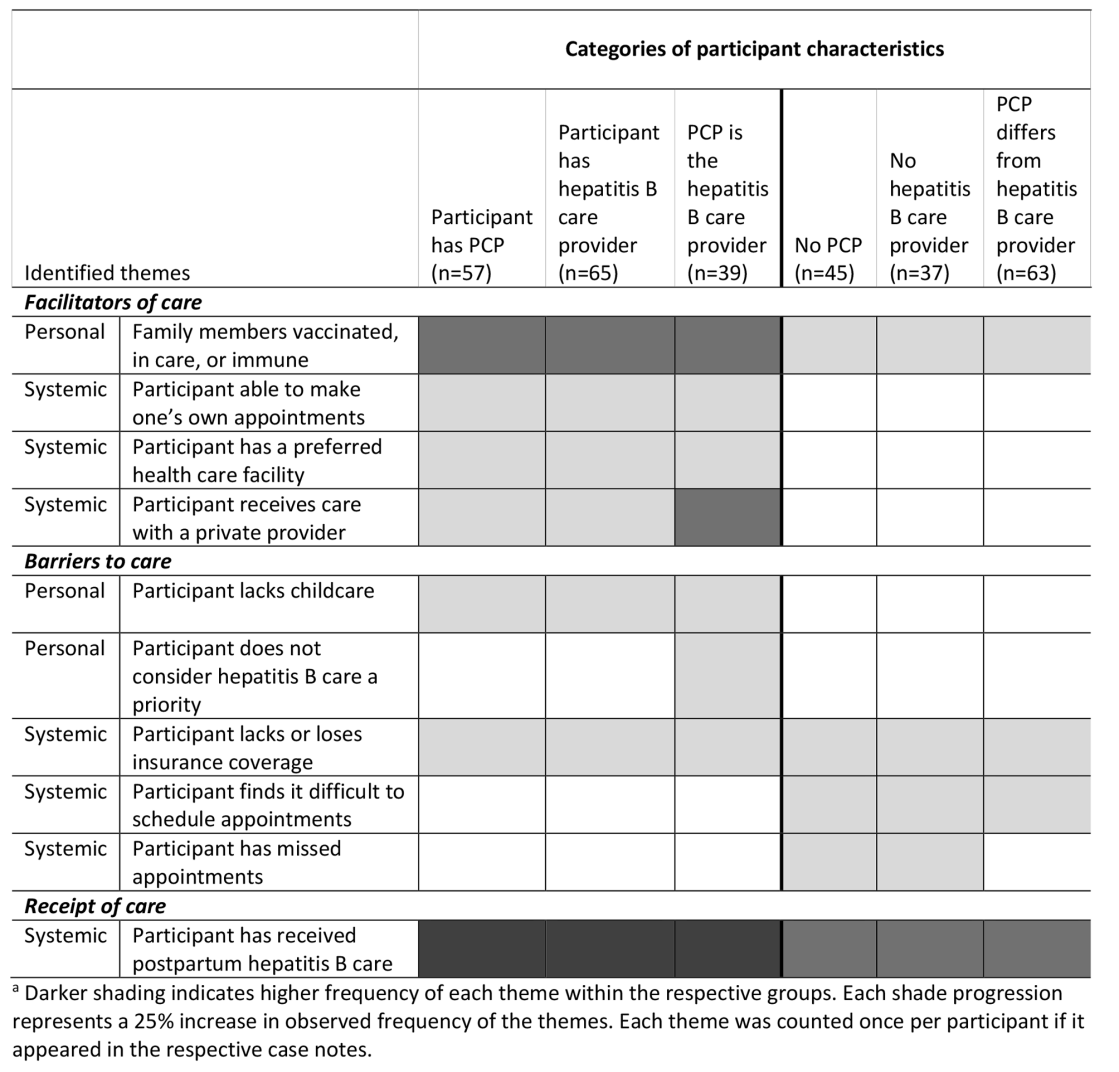
Heatmap illustrating frequently noted facilitators and barriers to postpartum hepatitis B care, by provider type.^a^

Based on our analysis, Fig. 1 summarizes the most prominent facilitators and barriers to care associated with having regular providers, as reported at initial assessment. As Fig. 1 illustrates, participants who had regular providers were more likely to note facilitators to care and to have successfully received hepatitis B care (with or without the patient navigator’s support), even though they were equally likely to experience disruptions in insurance coverage and experienced childcare challenges more frequently than participants who did not have a PCP and/or a hepatitis B care provider.

Some participants experienced significant barriers to care. First, 39 participants were noted as lacking insurance coverage or lost coverage during the intervention. Loss of insurance was strongly associated with missing scheduled appointments: 22 participants were noted to have missed appointments and, of these, 13 also reported losing health insurance coverage. Additionally, eight participants reported that their insurance did not fully cover the costs associated with their care and required prohibitive deductible and/or copay amounts. Fifteen participants lacked knowledge about what their insurance covered, when it terminated, how to indicate a PCP (a frequent pre-requisite for using the coverage), when referrals were needed, and how to check their eligibility or coverage status.

In response, patient navigators collaborated with the participants to call their insurers for more information. At times, this was a challenging experience, as insurance representatives abruptly disconnected calls due to patient privacy regulations once they learned that the caller was a patient navigator, despite receiving the participants’ consent to joint calls. Patient navigators then coached participants on how to call the insurers and request interpretation services, but often interpreters were not available and the participants could not establish communication with the insurers on their own. Conversely, some participants reported that they were in touch with dedicated representatives at their health insurance companies who spoke their primary languages. Unsurprisingly, those participants had a much easier time communicating with the insurance providers and did not need patient navigators’ support in this area.

Patient navigators addressed participants’ loss of insurance coverage by helping identify and make appointments with providers that offer low-cost or sliding fee scale services, namely FQHCs and public hospitals. Patient navigators gave instructions to participants about the process of applying for sliding fee scale designation and advised them about the types of documentation they would need to bring to appointments (e.g., proof of income, proof of address, proof of household composition). In a few cases (3), patient navigators connected participants with patient assistance programs offered by pharmaceutical companies to apply for free or low-cost hepatitis B medications. Patient navigators also advised participants who were at risk of losing insurance coverage that patient assistance programs might be an option for them.

Another barrier that patient navigators frequently addressed was the difficulty of scheduling appointments. This barrier was explicitly noted in 20 cases, though many more participants accepted patient navigators’ offers to make appointments, whether or not they experienced difficulties. Seven of the 20 received care in private hospitals, five in public hospitals, and one with a private provider; for the remainder, the type of provider was not noted. In some cases, patient navigators themselves found making appointments challenging, noting repeated attempts to reach schedulers at health care facilities, leaving multiple voicemails and emails and speaking with multiple staff members at facilities before appointments were confirmed. The task was made more complicated by participants’ schedule limitations, requests to reschedule appointments, and health insurance requirements. Making appointments sometimes posed a challenge for trained, English-speaking health care navigators illustrating the complexity of navigating the health care system in general and even more so for New Yorkers born outside of the U.S. living with hepatitis B who may not be proficient in English.

Only four participants were noted as having transportation difficulties as a barrier to attending appointments. For some, this meant that they did not know the NYC subway or bus system well and were unable to read English language signage on trains and buses; patient navigators explained travel routes to participants in detail in preparation for appointments. Others lived very far from the facilities where they had previously received hepatitis B care and asked patient navigators to identify providers closer to their homes.

**Fig. 2:**
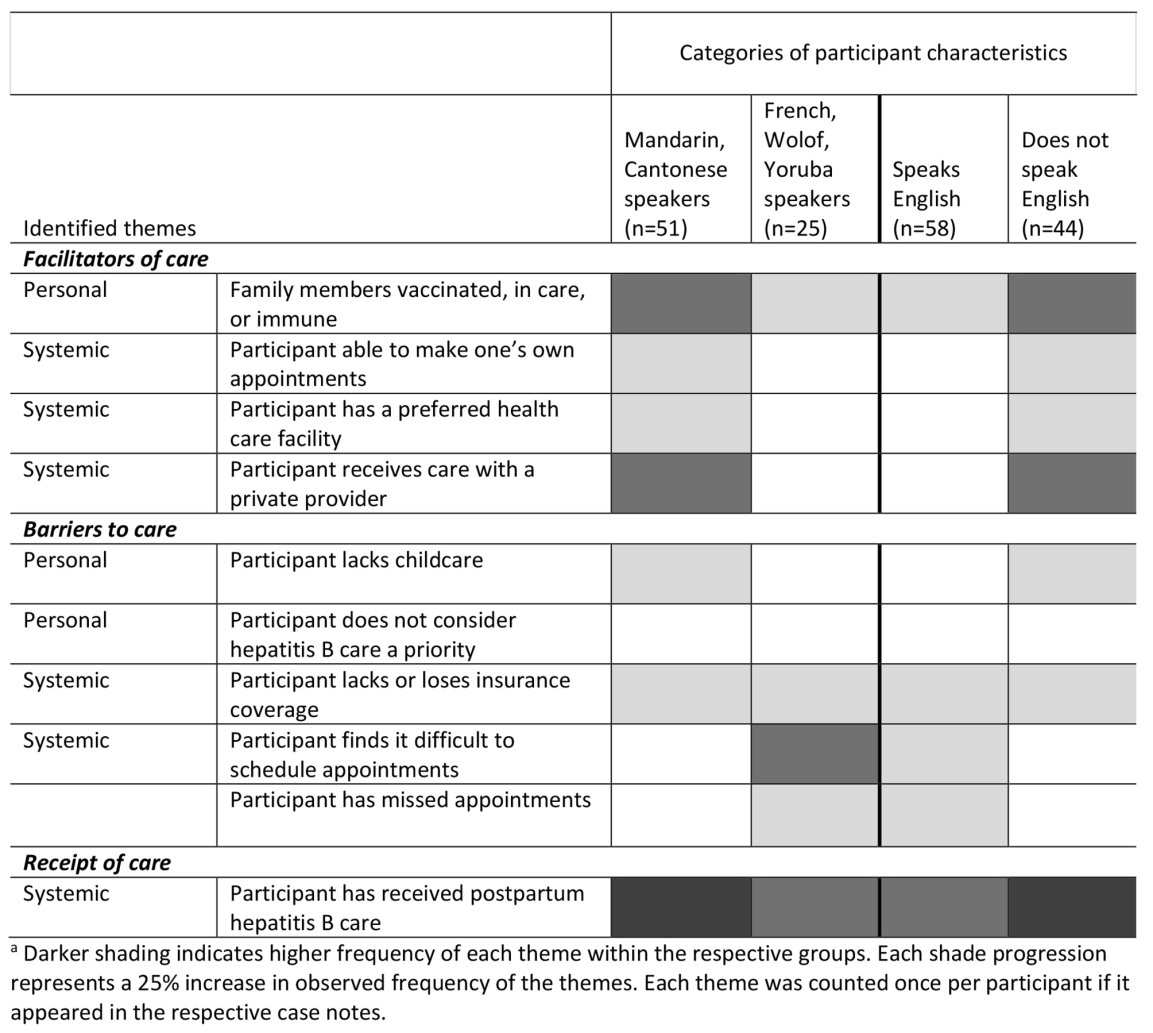
Heatmap illustrating frequently noted facilitators and barriers to postpartum hepatitis B care among language groups.^a^

Additionally, 14 participants reported poor communication with their providers. For example, they left appointments with unanswered questions about laboratory results and/or medication regimens or did not receive promised calls regarding their laboratory results. In some cases, communication difficulties stemmed from language barriers and the absence of interpreters, while in others, participants had difficulty understanding providers who spoke their languages. In response, patient navigators called providers to clarify information the participants had not understood and communicated the information back to the participants. They also encouraged participants to ask questions during future health care visits. Less frequently reported factors such as poor communication with providers and transportation difficulties were not included in the *Figs. 1 and 2* to draw focus to the most prominent facilitators and barriers in the analysis. It was not possible to compare trends in the less frequently mentioned factors influencing care across the categories of interest (provider status and language group) because of their low overall occurrence. Nonetheless, the additional facilitators and barriers are mentioned here because of their salience to the participants’ experiences with accessing hepatitis B care and the likelihood that some were under-recorded in the case notes.

Lastly, in the fall of 2018, near the end of the study period, the U.S. Department of Homeland Security announced a new version of the Public Charge Rule, which barred some immigrants from obtaining permanent residence if they used certain public benefits. The rule was enacted in August 2019 and halted in March 2021. Although the study period did not overlap with the enforcement of the Public Charge Rule, the anticipation of its enactment was a source of concern. As subsequent studies have found, the Public Charge Rule had a widespread negative effect among immigrant communities, keeping many from using safety net services even if their individual cases were not subject to the regulation [31]. In our sample, five participants chose to delay care because they did not want to apply for subsidized health insurance, Supplemental Nutrition Assistance Program (SNAP), and other benefits due to potential effects on their application for residency in the U.S.

### Facilitators and barriers to care by primary language group

We explored differences in facilitators and barriers to care based on primary language groups and whether participants may have experienced language barriers. Figure 2 summarizes the frequently noted facilitators and barriers to accessing hepatitis B care and receipt of care during the postpartum period among the language groups. As illustrated in Fig. 2, participants who reported that they did not speak English expressed more facilitators to care and fewer barriers to care than those who did speak English.^2^ As noted above, Mandarin- and Cantonese-speaking participants were less likely to report speaking English than French, Wolof, and Yoruba speakers. Importantly, Mandarin and Cantonese speakers were also more likely to have a hepatitis B care provider (χ^2^ = 20.8, p < .001), to have a PCP (χ^2^ = 34.7, p < .001), and for their PCP to be the hepatitis B care provider (p < .001, Fisher’s exact test) than French-, Wolof-, and Yoruba-speaking participants (Appendix C). In other words, our exploration of differences based on primary language and English proficiency led back to the same observation as above: the central importance of accessing care through a known provider.

In NYC neighborhoods with large East Asian immigrant communities, there are many private health care providers who speak Mandarin and/or Cantonese and treat hepatitis B.^3^ There are also several large FQHCs with on-site Chinese-speaking operators, patient navigators and providers who offer low-cost services and serve the Chinese immigrant community. Conversely, one of the patient navigators had difficulties finding health care providers who speak French, Wolof, or Yoruba and have focused services for the West African community. Participants from West Africa were more likely to be referred to hospitals, which the patient navigator selected based on location and whether they offer low-cost services to uninsured patients rather than for their cultural responsiveness or for their potential to become the participants’ primary care facility.

### Participants’ questions and concerns

As part of the intervention, patient navigators provided health education to participants using a script developed for the telephone-based patient navigation program. The script covered the basics of hepatitis B progression, vaccination, and liver health maintenance as well as transmission pathways and prevention. Patient navigators provided the option for participants to receive health education information verbally over the phone, to receive health promotion materials via email or text message, and to ask questions. The questions that participants asked and the concerns they shared were documented and analyzed as part of the case notes. The most commonly asked questions were about hepatitis B transmission, followed by medication regimens, disease progression, vaccination, and accessing low-cost care. Table 2 provides examples of the typical questions in each domain.

**Table 2:** Participants’ common questions about hepatitis B – New York City, July 1, 2016 – March 31, 2019

Question domain	Frequency in the case notes	Example
Hepatitis B transmission	16	Would hepatitis B spread to others and my newborn baby if I share chopsticks with them?
Medication regimen, hepatitis B blood test	12	Is hepatitis B medication harmful to the body in the long term?
Disease progression	12	Why did my viral load increase from around 300 last time to around 1000 this time?
Vaccination	12	My husband received the hepatitis B vaccine in China a long time ago. How can he check whether he still has immunity to hepatitis B?
Sliding fee/low-cost care	11	What is the cost of hepatitis B treatment? Can I get free hepatitis B medication if I become uninsured?
Nutrition, alternative medicine	7	What can I eat to make sure the virus stays low?
Community/family stigma	2	[Participant] would like husband to get tested, but also would like patient navigator to call husband and not disclose who referred him.
Employment discrimination	2	Can I work as a nurse or in the health care industry if I’m hepatitis B-positive?
Other health issues	1	What other health problems can hepatitis B lead to?

In response to concerns such as accessing sliding fee scale or low-cost services and vaccination effectiveness, patient navigators spoke with participants about low-cost hepatitis B care options and offered referrals for family members to check their vaccination status. For questions having to do with individual medical history and disease progression, such as the question about viral load increase in Table 2, they encouraged participants to ask their health care providers or directly supported that communication. The questions asked most frequently were often specific to the type of content covered in the telephone-based patient navigation program’s health education material.

## Discussion

Our analysis of the telephone-based patient navigation intervention case notes revealed useful context about participants’ experiences with accessing hepatitis B care. Our findings illustrated the many ways in which patient navigators supported participants, from communicating with health care providers to navigating health insurance eligibility. The relationships that patient navigators developed with participants, despite the limitations of a telephone-based patient navigation intervention, allowed them to respond to and strategize around participants’ individual challenges to accessing care. This is especially important to note as many patient navigation programs have had to shift from in-person to telephone-based services in the current context of the COVID-19 pandemic.

In addition, our findings reinforce the central importance of having a PCP and a preferred health care facility. Participants’ access to linguistically competent hepatitis B care in community-based primary care settings surfaced as a key factor in facilitating hepatitis B care. As mentioned in Fig. 2, compared to French, Wolof, and Yoruba speaking participants, Mandarin and Cantonese speakers were more likely to have a hepatitis B care provider, despite being less likely to report speaking English. This may be due to the widespread availability of Mandarin- and Cantonese-speaking providers in NYC and the likelihood of establishing a long-term relationship between participants and providers with the same cultural background. The intervention focused on connecting participants to hepatitis B care as soon as possible in the postpartum period and supporting their family members to access hepatitis B screening and care, but not on building a long-term comprehensive health care delivery plan. Based on our findings, the NYC DOHMH VHP’s telephone-based patient navigation program and similar patient navigation initiatives should consider expanding their goals to include helping participants establish a PCP and a medical home where they feel comfortable accessing care on an ongoing basis.

Our findings also shed light on the uncertain period following the loss of temporary state-sponsored medical insurance provided to pregnant people. While some participants were able to extend the temporary coverage and/or transition to other insurance types with minimal interruptions, for many the loss of temporary coverage presented a significant roadblock to continuing care. In the absence of universal health care, continued efforts by patient navigation interventions such as the NYC DOHMH VHP’s telephone-based patient navigation program are needed to help participants apply for and access the piecemeal low-cost, sliding fee scale options available. In June 2023, temporary NYS Medicaid coverage was extended to one year following childbirth; [32] however, our findings point to a need to advocate for NYS to extend temporary Medicaid coverage even further in order to allow postpartum individuals more time to navigate ongoing medical care for chronic conditions such as hepatitis B. In the absence of long-term insurance coverage options, it is also important to offer education about existing low-cost hepatitis B services and insurance coverage programs. Additionally, since health insurance in the United States is usually accessed through employer-sponsored coverage or through state-sponsored programs for low-income individuals and families, immigrant populations in the United States are less likely to have access to these coverage options due to their employment status and insurance eligibility restrictions that exclude undocumented individuals. Future studies may consider collecting data on employment status to measure the impact of employment on insurance coverage and health care access.

To further address the participants’ challenges, it appears that more thorough, multilingual health education materials with graphics and multimedia components, as well as the engagement of subject-matter specialists when designing materials directly in languages other than English, may be necessary. This is especially the case considering that some participants might have had questions later but refrained from asking during their conversations with patient navigators. The materials should communicate that participants can and should ask questions of their health care providers during appointments. Additionally, though there were few questions related to stigma and employment discrimination, it may be helpful to add information about patients’ rights, particularly as recent immigrants (such as the VHP’s patient navigation intervention participants) might not be aware of patient confidentiality and employment discrimination regulations and redress procedures in the U.S.

Finally, it is important to note a documented lack of access to appropriate translation services for people with limited English proficiency across the healthcare system, including hospitals, medical practices, and insurance companies. NYS could consider expanding their Patient Bill of Rights to apply to all insurers and other providers of medical care services to explicitly include access to interpretation services or, when not available, to allow patients to request that another person be present with them to support comprehension and enable them to ask questions and fully participate in the care planning process.

Our qualitative analysis corroborates findings of past studies that have identified facilitating factors and barriers as they relate to hepatitis B prevention and treatment. Qualitative and mixed methods research with immigrants from Africa [10, 16, 17, 25, 26] as well as East Asia [33] have highlighted the importance and continued need for culturally relevant hepatitis B education in prevention and treatment efforts. Though not explicitly named as a facilitator by participants in our analysis, likely due to the nature of the communication documented in the case notes, the importance of linguistically and culturally competent lay health workers to intervention success has been specifically acknowledged in extensive reviews of hepatitis B and C testing, linkage to care, treatment uptake and adherence initiatives [34, 35]. Some barriers identified in our analysis, such as lack of insurance, language and logistical challenges, have been acknowledged in some assessments of hepatitis B and C linkage to care initiatives among foreign-born adults in the U.S. [15, 36], but did not necessarily reflect the financial and cultural barriers expressed by foreign-born people living with hepatitis B in the NYC area [37].

However, our analysis fills an important gap in the scientific literature as there has been no prior research of this type focusing on new parents born outside of the U.S. and living in NYC, to our knowledge. Our research findings on facilitators and barriers deepen our understanding of how to improve timely delivery of hepatitis B care, with far-reaching impact for this population in large urban areas such as NYC.

An important limitation in this analysis is the variability of the case notes across patient navigators. Some patient navigators wrote more detailed notes than others; this variation was apparent even within the sample selected for completeness of notes. As such, we looked at the presence and relative frequency of themes, but we did not use the full dataset as a numeric denominator for comparison. For example, we were not able to state that a certain percentage of the 102 participants in the dataset experienced lack of childcare as a barrier to care because it is likely that the sparse notes available for some of the cases do not fully document the conversations between patient navigators and participants. In addition, it is likely that transportation issues were under-noted in the case notes and presented a barrier for more than four participants.

Relatedly, because we analyzed case notes rather than transcripts, we could not assess the nuances of cultural responsiveness practiced by patient navigators because the notes focused on the practicalities of following up with participants and making health care appointments. Patient navigators, originally from the same regions as many of the participants, communicated with participants in Mandarin, Cantonese, English, French, and Wolof and used telephone interpretation services to reach speakers of other languages. However, the notes offered little insight into the nuances of cultural responsiveness practiced in this intervention, e.g., how patient navigators built rapport with participants, how they referenced common cultural markers, how they communicated an understanding of the immigration experience. We therefore did not discuss cultural responsiveness as a characteristic of the intervention, beyond linguistic competency.

Per the protocol, patient navigators stopped taking notes in the study database at the conclusion of the study in March 2019. Some of the cases that we reviewed lasted past the study period. We did not have access to the full trajectory of these cases and could only report on what was documented in the study notes. Most cases in our dataset were closed by the end of the study period, either because the participants were in hepatitis B care and no longer needed support or because they had disengaged from the intervention.

Additionally, the study did not use randomization to create an intervention and standard of care group. Past research highlighted demographic differences between individuals who chose to participate in the intervention and those who did not [20]. As such, our case notes analysis is not generalizable to postpartum foreign-born people living with hepatitis B but contains lessons more specifically about the experiences of the subgroup of people who received patient navigation services.

Lastly, the outcomes of the program and the results of our previous study were shared with NYC hepatitis coalitions and committees, and nationwide healthcare summits that are addressing hepatitis B barriers among the affected immigrant population. Results from this study will also be shared internally within NYC DOHMH and externally with organizations serving this demographic.

## Conclusions

This qualitative analysis of case notes from a patient navigation intervention provides insight into the facilitating factors as well as the barriers to linkage to hepatitis B care encountered by parents born outside of the U.S. living in NYC. This research suggests that while there are numerous barriers at the personal and systemic levels, patient navigation programs such as the NYC DOHMH VHP’s program support people through hepatitis B care. These qualitative findings describe participants’ perspectives and experiences in detail and therefore serve as an important complement, not only to the intervention study evaluating the impact of the telephone-based patient navigation, but also to other epidemiologic research findings that may lack this valuable contextual information. This analysis can be leveraged to inform future program development to improve accessibility to care and health outcomes among people living with hepatitis B receiving patient navigation services.

### Electronic supplementary material

Below is the link to the electronic supplementary material.

Supplementary Material 1

## Data Availability

The data that support the findings of this study are available on request from the corresponding author [L.Y.T.], conditional on institutional approval. The data are not publicly available due to information on individual characteristics and health status contained therein that could potentially compromise participant privacy.

## List of abbreviations

DOHMH: Department of Health and Mental Hygiene
FQHC: Federally Qualified Health Center
HBsAg: Hepatitis B surface antigen
NYC: New York City
NYS: New York State
PCP: Primary Care Provider
PHBP: Perinatal Hepatitis B Prevention
SNAP: Supplemental Nutrition Assistance Program
U.S.: United States
VHP: Viral Hepatitis Program

## Footnotes

1 One of these two participants received care at a public hospital. The provider type for the other one was not noted.

2 These were individuals who indicated that they spoke English during the initial assessment, including those who spoke it as a primary language as well as those who spoke other primary languages. Patient navigators did not assess the participants’ level of English proficiency.

3 Patient navigators did not specifically document whether individual providers spoke the same primary languages as the participants.
