# Supplementary material for: Facilitators and barriers to accessing hepatitis B care in the postpartum period among foreign-born New Yorkers: a qualitative analysis of case notes
Source: BMC Public Health. 2024 Jan 8;24:116. doi: 10.1186/s12889-023-16971-3 (PMC10775572; doi:10.1186/s12889-023-16971-3)
Supplement: Supplementary file 1 — Supplementary Material 1 [file 12889_2023_16971_MOESM1_ESM.docx]

**Appendices**

**Appendix A. Semi-structured interview form.**

**Case Information**

NYC DOHMH Electronic Surveillance System Event ID:

Study ID:

Enrollment Date:

**Patient Demographic Information**

Patient Name:

Patient Address:

Patient Phone number(s)

Patient Email:

Permission to text: □ Yes □ No

Permission to email: □ Yes □ No

Primary and secondary language(s) spoken:

Speaks English: □ Yes □ No

Read English: □ Yes □ No

Best time/way to contact:

| **Part 1: Assess current engagement in hepatitis B medical care** |
| --- |
| **1. Do you have any form of health insurance?** □ Yes □ No  Insurance Type (if known): □ Medicaid □ Insurance for pregnant women (temporary Medicaid) □ Private Insurance □ HHC Options □ Does not have any insurance □ Medicare  Insurance Policy Name (if known):  Had insurance during pregnancy? □ Yes □ No  **2. Are you taking medication right now for your hepatitis B infection?** □ Yes □ No  Medication during pregnancy? □ Yes □ No  **3. Do you have a medical provider for hepatitis B and liver health care that you can make an appointment with?** □ Yes □ No  Provider name:  Provider location:  *This does NOT include the provider who monitors pregnancy, or a primary care provider, unless this provider also checks their liver regularly.*  **4. When was your most recent appointment with this provider?**  Most recent medical appointment date:  **5. When is your next appointment with this provider?**  Do they have an appointment scheduled already? □ Yes □ No  Next appointment date:  If answer is “NO”, then schedule the next appointment.  **6.** **Brief Health Literacy Assessment - How easy or hard is it to fill out medical forms by yourself?**  □ Very Hard □ Somewhat Hard □ Easy □ Very Easy |
| **Part 2: Assess barriers to obtaining care and following provider recommendations for treatment and follow-up** |
| **7. Who is the primary care doctor that you see for check-ups or when you get sick (i.e., family practitioner or general practitioner, internist, OB/GYN)?**  □ Has a doctor □ Does not have a doctor  Provider/clinic name:  Provider location:  Date of last visit:  **8. Some people are concerned about the cost of medical exams, tests, or medications. Do you have any questions or concerns about these costs?** □ Yes □ No, Notes:  **9. Some people have other personal, family or other challenges that seem more important than taking time out to follow up on their own hepatitis B care. Are there things that make it hard to follow up on your medical care?** □ Yes □ No  If the client answers ‘Yes’ to this question, ask if they can tell you more about these problems:  Personal concerns □ Yes □ No, Notes:  Public benefits concerns □ Yes □ No, Notes:  **10. Is there anything that would help?** □ Yes □ No, Notes:  **11. Can I help schedule an appointment with a medical provider for you to check the health of liver and monitor hepatitis B?** □ Yes □ No |
| **Part 3: Basic Health Counseling and Contact information** |
| **12. Do you have any general questions about hepatitis B that you would like to be answered?**  □ Yes □ No, Notes:  **13. Provide basic hepatitis B and liver health education.** Notes:  **14. Do you have family, friends, or household members who may need hepatitis B screening, vaccination, or evaluation?** □ Yes □ No, Notes: |
| **Part 4: Follow up planning and linkage to care** |
| **15. Summarize linkage to care plan (e.g., next steps) for patient. Offer to provide reminders, create a follow-up plan as appropriate.**  Linkage to care plan summary:  **16. Do you need any additional referrals or resources in your community?** □ Yes □ No  Referral to (list provider/organization name/location):  **17. Close out call and thank the participant. Once again, confirm they have your contact information & know how to get in touch with you.**  **18.** **Tell Patient: “I am going to call you on THIS DATE to discuss your next appointment, is that okay with you?”**  Follow up permission from patient: □ Yes □ No  Date that you will follow up with patient: |
| **Part 5: Following up with patient** |
| **19. Follow up notes:**  **20. Linkage to care activity:** □ Scheduled appointment for patient □ Patient will contact current provider □ Referral provided and accepted □ Declined referral □ None: In care  Total number of encounters for patient:  Total hours and minutes spent for patient:  **21. Did patient attend appointment?** □ Attended □ Declined □ No show □ Unknown  Date of attended appointment:  Name of provider:  **22. Changed medication status:** □ Stopped medication-advised by provider □ Stopped medication on own □ Started medication for the first time □ restarted medication  **23. Changed insurance status:** □ insurance type change □ Lost insurance □ Gained insurance |

**Appendix B. Codebook of identified themes and categories used to analyze case notes.**

*Personal Facilitators and Barriers to Care*

- Financial facilitators
  - Out of pocket price for hepatitis B care is accessible for participant
  - Participant is able to find affordable medication/services
- Financial barriers
  - Participant lacks or loses insurance coverage
  - Participant’s insurance does not fully cover the costs of care (participant cannot afford copays, deductibles)
  - Participant lacks knowledge about insurance coverage (e.g., whether insurance is active, how much are the copay amounts)
  - Participant’s work schedule prevents them from being able to attend appointments
- Family facilitators
  - Participant has family support to get care (e.g., accompaniment to appointments, childcare)
  - Participant refers contacts for hepatitis B testing/care
  - Participant’s family members are vaccinated, in care, or have immune control
- Family barriers
  - Participant lacks childcare
  - Participant lacks other family supports
  - Family members have not been tested, are not in care for hepatitis B
- Other barriers
  - Participant has immigration-related concerns pertinent to the Public Charge Rule
  - Participant lacks stable housing
  - Participant lacks transportation options and/or lives far away from their provider
  - Participant does not consider hepatitis B care a priority

*Systemic Facilitators and Barriers to Care*

- Type of health care facility where participants receive care
  - Private provider
  - Non-public hospital
  - FQHC
  - Public hospital
- Health care system facilitators
  - Participant is able to make one’s own appointments
  - Participant has received postpartum hepatitis B care
  - Participant has had positive experiences with provider or healthcare system (e.g., positive feedback about quality of care, communication)
  - Participant has a preferred health care facility
- Health care system barriers
  - Participant experiences poor communication with provider, language barriers
  - Participant finds it difficult to schedule appointments
  - Participant has missed appointments
  - Participant has never before received hepatitis B care (during pregnancy or otherwise)
  - Participant has trouble accessing care for other health conditions

*Program Experience*

- Experience with the NYC DOHMH VHP’s telephone-based patient navigation intervention
  - Patient navigator made appointment(s) for participant
  - Patient navigator addressed insurance issues with participant
  - Patient navigator facilitated connection with a patient assistance program (for which the participant can apply via a provider or pharmacy)
  - Patient navigator assisted in finding a hepatitis B care provider
  - Patient navigator helped establish communication with a provider
  - Patient navigator helped resolve participant’s questions, doubts
  - Participant disengaged from program (lost to follow-up)
- Participants’ questions or concerns
  - Transmission
  - Medication regimen/side effects/treatment/lab work
  - Disease progression/viral load/symptoms
  - Nutrition/diet/vitamins/alternative medicine
  - Vaccination
  - Other health issues caused by hepatitis B
  - Need for routine follow-up even in absence of symptoms
  - Stigma from family/friends/community
  - Employment discrimination due to hepatitis B status
  - How to access sliding fee/low-cost care

**Appendix C.**

| **Table B1. Sociodemographic and health care-related characteristics of foreign-born participants interviewed to assess facilitators and barriers in accessing hepatitis B care in the postpartum period – New York City, July 1, 2016 – March 31, 2019.** | | | | |
| --- | --- | --- | --- | --- |
|  | **Mandarin and Cantonese speakers (n=51)** | **French, Wolof, and Yoruba speakers (n=25)** | **English speakers (n=26)** | **Total (n=102)** |
| Reports speaking English | 11 (21.6%) | 21 (84.0%) | 26 (100%) | 58 (56.9%) |
| ***Borough of residence at initial assessment*** | | | | |
| Brooklyn | 39 (76.5%) | 4 (16.0%) | 6 (23.1%) | 49 (48.0%) |
| Bronx | 0 (0%) | 13 (52.0%) | 9 (34.6%) | 22 (21.6%) |
| Queens | 9 (17.6%) | 4 (16.0%) | 7 (26.9%) | 20 (19.6%) |
| Manhattan | 3 (5.9%) | 4 (16.0%) | 2 (7.7%) | 9 (8.8%) |
| Staten Island | 0 (0%) | 0 (0%) | 2 (7.7%) | 2 (2.0%) |
| ***Insurance status at initial assessment*** | | | | |
| Insured | 31 (60.8%) | 20 (80.0%) | 21 (80.8%) | 72 (70.6%) |
| Medicaid  Private insurance | 23 (74.2%)  8 (25.8%) | 18 (90.0%)  2 (10.0%) | 14 (66.7%)  7 (33.3%) | 55 (76.4%)  17 (23.6%) |
| Temporarily insured | 16 (31.4%) | 4 (16.0%) | 3 (11.5%) | 23 (22.5%) |
| Uninsured | 4 (7.8%) | 1 (4.0%) | 2 (7.7%) | 7 (6.9%) |
| ***Health care provider status at initial assessment*** | | | | |
| Has provider for hepatitis B care | 43 (84.3%) | 8 (32.0%) | 14 (53.8%) | 65 (63.7%) |
| Has primary care provider (PCP) | 45 (88.2%) | 5 (20.0%) | 7 (26.9%) | 57 (55.9%) |
| PCP is the hepatitis B care provider | 35 (68.6%) | 3 (12.0%) | 1 (3.8%) | 39 (38.2%) |
